# A Rare Case of Pulmonary and Gastrointestinal Mucormycosis Due to *Rhizopus* spp. in a Child with Chronic Granulomatous Disease

**DOI:** 10.3390/idr14040062

**Published:** 2022-08-08

**Authors:** Nnennaya U. Opara

**Affiliations:** 1 Department of Emergency Medicine, Charleston Area Medical Center, Institute for Academic Medicine, Charleston, WV 25304, USA; nnennaya.opara@camc.org; 2 Department of Medicine, Alliance Hospital, Abuja 900247, Nigeria; 3 Health Administration, University of Phoenix, Phoenix, AZ 85040, USA

**Keywords:** mucormycosis, *Rhizopus* spp., pediatric, gastrointestinal, pulmonary, CGD, aspergillosis

## Abstract

Mucormycosis is a rare but serious fungal infection caused by a mold family known as the Mucorales. These fungi exist throughout the environment, especially in the soil, leaves, compost piles, or decaying woods. Humans contract mucormycosis by coming in contact with the spores from fungus either by inhalation or through cuts on the skin. The population at risk for this life-threatening infection includes diabetes mellitus patients, cancer patients, premature infants, burn patients, and immunocompromised patients. The fungi that most commonly cause mucormycosis are the *Rhizopus* species, and the least represented are *Apophysomyces* species. Common clinical manifestations of mucormycosis include pulmonary, cutaneous, rhinocerebral, and gastrointestinal mucormycosis. Cases of lung mucormycosis are often misdiagnosed because of non-specific clinical symptoms and radiological features, and in many cases, have been diagnosed as *aspergillosis* due to similarities in signs, symptoms, and imaging presentation of the lungs. We present a pediatric case of a 6-year-old from Togo who presented to our hospital in Nigeria with dyspnea, fever, and abdominal pain of five-day duration. The child’s symptoms began 6-months prior, with dry cough, fever, fatigue, and chest pain and abdominal pain. The hospital in Togo where he lived suspected infection with tuberculosis (TB) despite a false-positive Mantoux test and negative chest X-ray. He was initially treated for TB with Isoniazid and vitamin B6 and was discharged home. Six months later, his symptoms have not improved, but became more severe with high grade fever 40 °C (oral reading), anorexia, fatigue, tachypnea, abdominal distention, and cough. The patient was immediately referred to our hospital in Abuja, Nigeria where more specific tests were ordered. He was eventually diagnosed with chronic granulomatous disease induced pulmonary and gastrointestinal (GI) mucormycosis due to *Rhizopus* spp. In this report, we discuss an unusual clinical presentation of an infection caused by *Rhizopus* spp., its management, and outcomes in a child with chronic granulomatous disease (CGD).

## 1. Introduction

*Rhizopus* species, one of the most common types of mucormycetes that cause mucormycosis, is a rare but life-threatening fungal infection that primarily affects immunocompromised humans, with an estimated mortality rate of 23–100% [1]. Humans contract mucormycosis through contact with the fungal spores in the environment. The pulmonary form of the infection can occur after a person inhales the spores from the air. Cutaneous infection occurs through cuts or scrapes on the skin, and GI form through ingestion of the fungal spore. Mucormycosis is not spread from person to person or between animals and humans. Patients commonly affected include people with diabetes, patients on chemotherapy, and patients diagnosed with acquired or inherited immunodeficiency syndrome. *Rhizopus* spp. was first discovered in Germany by Furbinger in 1876 on a deceased patient who died of cancer and in whom the right lung showed a hemorrhagic infarct with fungal hyphae and sporangia [2]. Mucorales fungi are the second most common mold pathogens after Aspergillus, causing invasive fungal infection in patients receiving immunosuppressants and immunocompromised conditions [3]. The incidence of mucormycosis has also increased among diabetic ketoacidosis patients, particularly infections caused by Rhizopus oryzae infections as these fungi produce the enzyme called ketoreductase, which enables them to metabolize the patient’s ketone bodies for their growth [4], thus, making diabetes melittus the most typical risk factor worldwide. Clinical manifestation of mucormycosis infection depends on the organs harboring the fungus; in the case of pulmonary mucormycosis, common presenting symptoms include cough, fever, chest pain, and tachypnea. Gastrointestinal mucormycosis will present with abdominal pain, nausea, vomiting, and GI bleeding. We present a rare case of pulmonary and gastrointestinal Rhizopus infection in a child with an underlying immunocompromised condition.

## 2. Case Presentation

A 6-year-old boy from Togo presented to our hospital in Abuja, Nigeria, with dyspnea of five-day duration. Six months before this visit, he complained of abdominal pain localized in the umbilical region. His mother described the abdominal pain as intermittent, non-radiating, and with no aggravating or relieving factors. The abdominal pain was accompanied with a high-grade fever of 38.9 °C (underarm reading), and abdominal distention. He was initially managed at a regional hospital in Lomé (a city in Togo), where a CT scan revealed a mass in the left kidney, and he underwent abdominal paracentesis. At the same regional hospital, a biopsy of the mass was performed, which revealed fibrocollagenous tissue with granulomatous inflammation and caseous necrosis. Tuberculosis (TB) suspicion was raised, which required a chest X-ray and Mantoux test. The Mantoux test was false-positive due to previous Bacillus Calmette–Guerin (BCG) immunization, and the chest X-ray was negative for a mass lesion. The regional hospitalist prescribed a 6-month course of isoniazid plus vitamin B6 and discharged him to recover from home. His past medical history was positive for five-lifetime hospital admissions, four within six months of the current presentation. There was no exposure to blood transfusion, immunosuppressive drugs, and no history of surgery. Pregnancy was uneventful, with spontaneous vaginal delivery at full-term, and with low birth weight at 2.7 kg (6 pounds). The neonatal period was uneventful. Immunization was up to date, and the patient received the BCG vaccine. The patient’s development was tracked at the 50th percentile for height and at 25th percentile for weight. There was no family history of TB, HIV, diabetes, or CGD. The patient was living with his parents and four siblings in an old, wooded house with poor sanitation and close to lakes.

Though the patient completed the 6-month course of isoniazid, his condition continued to deteriorate with the recurrence of abdominal pain and distension associated with dyspnea. On examination in our hospital, the patient had marked tachypnea with respiration rate at 50 breaths per minute, PO_2_ at 80% in room air, a heart rate of 148 beats per minute, and blood pressure of 99/70 mmHg. Abdomen exam: distended abdomen with visible veins; girth measures at 68 cm; liver size at 7 cm below costal margin; fluid waves on abdominal percussion; and periumbilical pain. Chest examination showed decreased tactile fremitus and vocal resonance on the right chest. Presence of dullness on percussion with and absent breath sounds on the right lung. The patient was conscious but irritable, oriented to time, person, and place. The patient’s Body Mass Index (BMI) was 13 kg/m^2^ (underweight at the 2nd percentile). The patient looked cachectic; skin appeared pale, febrile, and absent clubbing of digits. Oral examination revealed excessive thrush.

## 3. Investigation and Management

**Figure 1:**
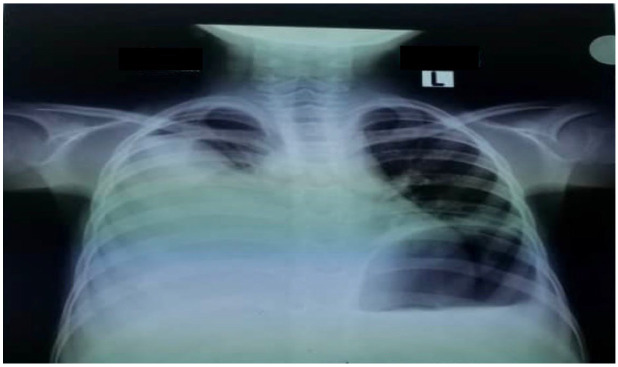
Chest X-ray of the patient showing right pleural effusion.

The patient was admitted to the pediatric intensive care unit (PICU) because of tachypnea, hypoxemia, and subcostal retraction. An urgent chest X-ray revealed a right pleural effusion (Figure 1).

**Figure 2:**
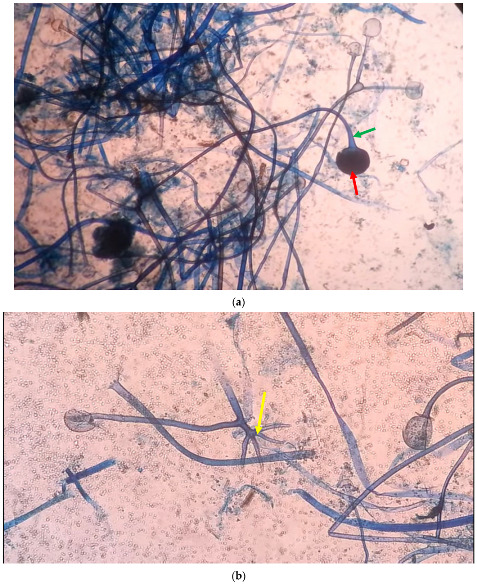
(**a**)**.** LPCB mount of *Rhizopus* in the lung aspirate of the patient showing sporangiospore (green arrow) and sporangium (red arrow) of a *Rhizopus* spp. (**b**). LPCB mount of *Rhizopus* in the renal cyst aspirate of the patient showing rhizoid (yellow arrow) of a *Rhizopus* spp.

Thoracocentesis was performed and was evident for chylothorax (exudative pleural effusion). Analysis of the pleural aspirate contents: total cholesterol 50 mg/dL, triglyceride 159 mg/dL (normal < 150), pleural protein ratio at 0.8, and lactate dehydrogenase (LDH) ratio 0.7. Direct microscopic examination of lung aspirate with lactic acid phenol cotton blue (LPCB) stain revealed nonseptate filamentous hyphae with branching sporangiophores in clusters and a round sporangium at the tip indicative of *Rhizopus* spp. (Figure 2a,b).

He received 5–8 L/min of 100% oxygen with a mask for respiratory distress, and his condition improved without requiring intubation at an oxygen saturation rate of 97%. He was discharged from the PICU to the general ward for continuous medical treatment on day three. Serology tests for hepatitis B and C were negative. A QuantiFERON-TB Gold was negative. Initial complete blood count revealed increased white cell count (neutrophilia at 9.12 × 10^9^/L; normal range 2.0–7.0 × 10^9^/L, and eosinophilia at 0.7 × 10^9^/L; normal range 0.04–0.4 × 10^9^/L) and urea 7.8 mmol/L, with decreased uric acid level 142 micromol/L; normal range 155–357 micromol/L, total serum protein 48 g/L; normal range 63–82 g/L, albumin 18 g/L; normal range 35–50 g/L, sodium level 130 mEQ/L; normal range 135–150 mEQ/L, Hemoglobin 8.7 g/dL; normal range for age 13.5–15 g/dL, and creatinine 52 mmol/L; normal range 62–106 mmol/L. Other lab results were unremarkable.

**Figure 3:**
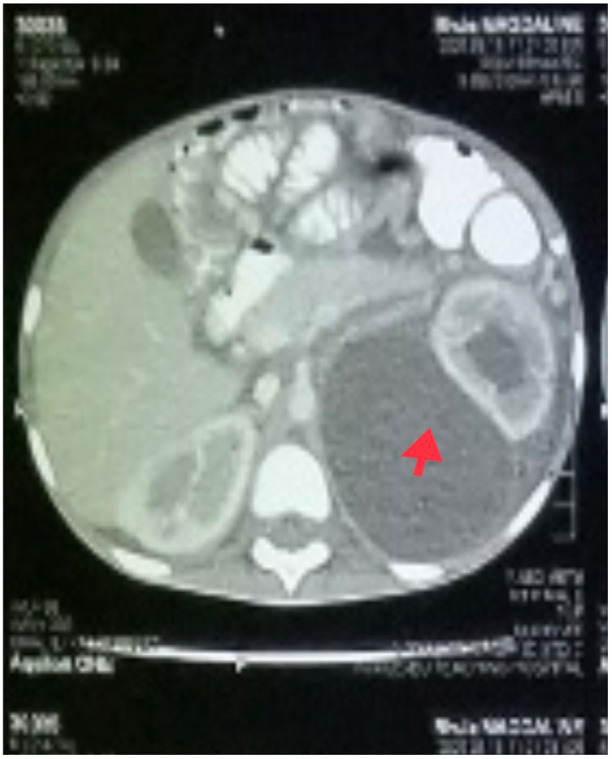
A cross-sectional view of the abdominal CT scan showing large left renal cyst (red arrow) with hydronephrosis.

In addition to starting broad-spectrum antibiotics (IV ceftriaxone 1 g daily and vancomycin 40 mg/kg daily) for suspicion of bacteria coinfection and IV electrolyte replacement and fluid resuscitation with normal saline infusion, an abdominal CT scan was performed, which revealed a large cystic mass (possibly a urinoma) on the left kidney with hydronephrosis and moderate ascites (Figure 3).

The mass was aspirated using an ultrasound-guided technique under general anesthesia. The aspirate showed chylous ascites, and the histopathology result of a fine needle biopsy of the left renal tissue of the mass lesion showed evidence of *Rhizopus* spp. Following the procedure, the patient continued to have pains not relieved with 250 mg acetaminophen every eight hours and was administered 200 microgram/kg of oral morphine TID. The patient was also started on 100 mg BID of itraconazole.

On the fifth day after admission (three days after PICU discharge), the patient developed new pulmonary effusion, cervical lymphadenopathy, hypoalbuminemia, and fever. Thoracentesis was performed, which drained milky turbid fluid. The patient’s echocardiography was normal. Histopathology of cervical lymph node biopsy revealed lymphocyte aggregation, diffuse granulomatous inflammation with eosinophils surrounded by numerous multinucleated giant cells, and pigmented epithelioid macrophages. Within the giant cells were fungi in the cytoplasm. Special staining with periodic acid Schiff (PAS) showed magenta staining of the fungal cell wall in the cytoplasm of the multinucleated giant cells. The patient’s blood sample was sent for a specialized laboratory testing with dihydrorhodamine 123 (DHR) to test the oxidative functional activities of macrophages of which returned a non-fluoresce DHR. He was subsequently diagnosed to have chronic granulomatous disease. The patient received liposomal amphotericin B at a dose of 7 mg/kg/day, then increased to 10 mg/kg/day. Interferon gamma was added to treatment with a chest tube for drainage.

**Figure 4:**
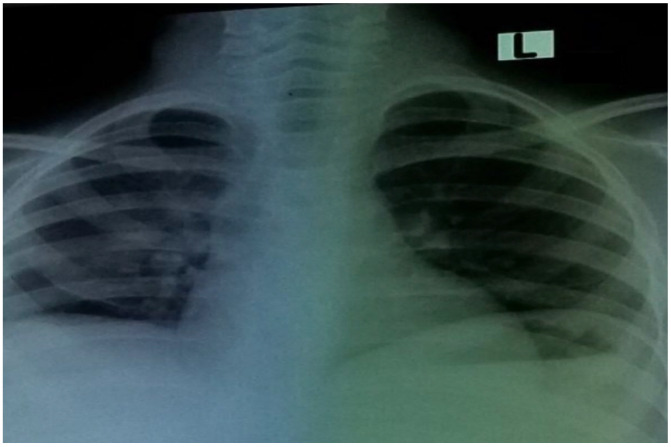
Chest X-ray of patient showing resolution of right pleural effusion on hospital admission day 7.

On day seven following admission, the patient’s overall health improved, and the chest X-ray showed effusion improvement (Figure 4).

His parents were informed about their child’s health condition and his need for a lifelong prophylaxis medication with antibiotics (trimethoprim and sulfamethoxazole combination for bacterial infections and itraconazole for fungal infections). The patient was also scheduled for periodic interferon-gamma injections to help boost the immune system. The child continued to improve his health condition, and he was discharged home to his parents on day 10.

## 4. Discussion

Chronic granulomatous disease (CGD) is a rare inherited immunodeficiency condition in which phagocyte (a form of white blood cell) does not work correctly. The disease is caused by a genetic mutation in genes encoding the phagocyte nicotinamide adenine dinucleotide phosphate oxidase, resulting in the defective generation of reactive oxygen species, thus, a person becomes vulnerable to life-threatening infections caused by catalase-positive bacteria and fungi. Patients with CGD are generally diagnosed their childhood period, but some people may not be aware that they have CGD until adulthood.

A recent study showed that the most common non-*Aspergillus* fungal infections in patients with CGD were due to *Rhizopus* spp. and *Trichosporon* spp. with the lungs as the most affected organs [5]. *Rhizopus* spp. are considered opportunistic pathogens because they require a breach in the immune defenses, particularly in the phagocytic function to initiate infections. In healthy individuals, macrophages and neutrophils protect against infection by secreting enzymes that neutralize the virulence factors of any microbe, a process known as phagocytosis and oxidative killing of fungal spores (in the case of mucormycosis) [6]. In our patient, the mode of transmission was very likely by inhalation as the hygienic conditions of his home was very poor and unhealthy, and with decaying wood in the house created a favorable condition for Mucorales growth.

Typical clinical presentation of mucormycosis consists of rhinocerebral (39%), pulmonary (24%), and disseminated (15%) [7]. The gastrointestinal (GI) form of mucormycosis is sporadic. There are two main clinical entities described by a study that categorizes the cause of death due to GI mucormycosis into (a) a non-invasive mucormycosis (superficial) of the gut without invasion of the blood vessels to having a good survival rate, or (b) an invasive mucormycosis of the gut with invasion of blood vessels with a 100% mortality [8].

In a review of 158 pediatric cases of mucormycosis published in the literature since 1939, the authors all discovered that 21% of the time, the infections were localized in the gastrointestinal tract [9]. In those cases, GI mucormycosis was associated with prematurity with a 100% mortality rate and no immunodeficiencies were noted among these children. However, in another study by Mooney and Wanger, GI mucormycosis in children were linked to underlying CGD, especially among children without any predisposing factors [10]. The most significant finding in these two studies is that GI mucormycosis, though rare, is more common in children than adults. Moreover, prematurity seems to be a significant factor in developing GI mucormycosis [9]. In addition, the combination of low birth weight (as seen in our patient birth record), immaturity of the immune system in neonates, and immaturity of the Gut-associated lymphoid tissue (GALT) system in young children is a predisposing factor for GI mucormycosis among these age groups. There have been other pediatric cases of disseminated mucormycosis in pediatric cancer patients in which a combination therapy of triazole and amphotericin B was used for treatment, and it decreased the mortality rates from mucormycosis in these patients’ population by 80% [11].

The most effective treatment for mucormycosis (*Rhizopus* spp.) is amphotericin B [6]. Other antifungal agents such as itraconazole, voriconazole, or fluconazole can be used for the same purpose. However, they are not as effective as amphotericin B (as was seen in the management of patients with pulmonary and GI mucormycosis who failed to make a full recovery on itraconazole but made a full recovery on the addition of amphotericin B). In those cases with bone involvement, surgery may be necessary [12]. Interferon-gamma has also proved effective in the prophylaxis against severe bacterial and fungal infections, particularly in patients with CGD [13]. Interferon-gamma can also be added to the treatment regimen for patients with pulmonary and GI mucormycosis. However, the mechanism of action describing its effect on improving neutrophilic functions remains under investigation.

## 5. Conclusions

Pulmonary and GI mucormycosis due to *Rhizopus* spp. in children can be fatal when not promptly diagnosed and treated. In immunocompromised patients, the disease is typically progressive and frequently fatal. Initial treatment should constitute a starting dose of liposomal amphotericin B (LAmB) dosed at 5 mg/kg daily (without CNS involvement) or 10 mg/kg daily in the case of central nervous system (CNS) involvement. Although for our patient, we administered itraconazole as the initial medication, this was due to his poor overall health condition (malnourished, severely underweight, and abnormal lab results) while replacing nutrients and fluid volume loss. As the patient became well rehydrated and nourished, LAmB was later introduced to his treatment regimen which improved his health condition. It is equally important to note that posaconazole is also a good alternative to LAmB in the absence of CNS involvement and if renal toxicity develops or when a patient does not tolerate LAmB due to unexplained reasons. Antifungal therapy should be continued for patients with *Rhizopus* spp. infection until clinical resolution of symptoms, and continuous monitoring of patients after discontinuation of treatment is also necessary. When examining children presenting with symptoms of infectious disease, clinicians must obtain an extensive medical history and living conditions. Our patient history of frequent hospitalization prompted an immunology test, leading to the diagnosis of CGD. Currently, there is no definitive treatment for CGD, and no cure or treatment that helps children with this type of disease process. However, continuous antibiotics and antifungal medications as prophylaxis against bacterial and fungal infections may help in preventing complications from CGD. Additionally, interferon gamma-1b injections (Actimmune) 0.5 mL single dose three times per week, may also be used preventatively to boost immune cells production. Our patient continues to thrive and has not had any hospitalization in the past 14 months following his discharge from the hospital. This further suggests that mucormycosis in immunocompromised patients can be effectively managed medically without surgery and that surgical intervention should only be reserved for disseminated mucormycosis involving the bones.

## Data Availability

Not applicable.
